# An electronic portal image device (EPID)‐based multiplatform rapid daily LINAC QA tool

**DOI:** 10.1002/acm2.13055

**Published:** 2021-01-07

**Authors:** Yangguang Ma, Xuemin Wang, Rizhen Mai, Tao Wang, Yuntong Pei, Shuaipeng Liu, Yuexin Guo

**Affiliations:** ^1^ Department of Radiation Oncology The First Affiliated Hospital of Zhengzhou University Zhengzhou China; ^2^ Department of Radiotherapy Hospital Unit Radiation Therapy Shaanxi Provincial Tumor Hospital Xi’an China; ^3^ Department of Medical Equipment The First Affiliated Hospital of Zhengzhou University Zhengzhou China

**Keywords:** electronic portal image device, quality assurance, quick daily check

## Abstract

**Purpose:**

To develop an efficient and economic daily quality research tool (DQRT) for daily check of multiplatform linear accelerators (LINACs) with flattening filter (FF) and flattening filter‐free (FFF) photon beams by using an Electronic Portal Image Device (EPID).

**Materials and Methods:**

After EPID calibration, the monitored parameters were analyzed from a 10 cm × 10 cm open and 60° wedge portal images measured by the EPID with 100 MU exposure. Next, the repeatability of the EPID position accuracy, long‐term stability, and linearity between image gray value and exposure were verified. Output and beam quality stability of the 6‐MV FF and FFF beams measured by DQRT with the introduced setup errors of EPID were also surveyed. Besides, some test results obtained by DQRT were compared with those measured by FC65‐G and Matrixx. At last, the tool was evaluated on three LINACs (Synergy, VersaHD, TrueBeam) for 2 months with two popular commercial QA tools as references.

**Results:**

There are no differences between repeatability tests for all monitored parameters. Image grayscale values obtained by EPID and exposure show good linearity. Either 6 MV FF or FFF photon beam shows minimal impact to the results. The differences between FC65‐G, Matrixx and DQRT results are negligible. Monitor results of the two commercial tools are consistent with the DQRT results collected during the 2‐month period.

**Conclusion:**

With a shorter time and procedure, the DQRT is useful to daily QA works of LINACs, producing a QA result quality similarly to or more better than the traditional tools and giving richer contents to the QA results. For hospitals with limited QA time window available or lack of funding to purchase commercial QA tools, the proposed DQRT can provide an alternative and economic approach to accomplish the task of daily QA for LINACs.

## 1 INTRODUCTION

Daily Quality Assurance (QA) is the most frequently performed procedure among all the QA procedures on linear accelerator (LINACs) and has a direct impact on the performance of the IMRT procedure. Existing daily QA methods are usually time‐consuming. For metropolitan hospitals in China, one of the major challenges for daily LINAC QA is the limited time window available, since most LINACs are overloaded with cancer patients: Taking the LINACs at our hospital as example, the average number of procedures performed per day is 90–120 per system, compared with 25–50 in Europe and 13–25 in the United States.^1^, ^2^, ^3^ Therefore, there is a strong clinical need to develop a more efficient and cost‐effective QA solution.

EPID has been introduced to LINAC systems since the early 1980s.^4^ It was initially developed for verifying the patient position and was later applied to LINAC QA.^5^, ^6^, ^7^, ^8^, ^9^, ^10^, ^11^, ^12^ Compared with films and other QA devices, EPID has two major advantages^13^, ^14^, ^15^: first, since EPID is integrated with the LINAC gantry, the QA procedure can be setup more quickly; second, the EPID data are in a digital form, which greatly facilitates the postprocessing, transfer, analysis, and storage of the data. Unlike the earlier generations of EPIDs, recent advances in the flat panel detector technology have enabled EPID to have sufficient spatial resolution for the purpose of LINAC QA. Taking the amorphous‐silicon (a‐Si) flat panel detector (PerkinElmer Inc., Santa Clara, CA) equipped by the iViewGT system (Elekta Oncology Systems, Crawley, UK) as an example, it has 1024 × 1024 detector pixels, each pixel with an isotropic size of 0.25 mm at the isocenter of the LINAC gantry. Hence the detector provides a total field of view of 40 cm × 40 cm.

The purpose of this work is to develop and assess a rapid EPID‐based multiplatform daily LINAC QA tool to meet the imperative clinical need for efficient and cost‐effective daily LINAC QA procedure. Note that using EPID for daily LINAC QA is not a new concept, as several previous works have been reported over the past decades, both for photon beams^16^, ^17^, ^18^, ^19^ and electron beams.^12^, ^20^, ^21^ For example, Clivio et al.^22^ and Michael et al.^23^ described a LINAC vendor‐provided QA tool (Machine Performance Check, MPC) which is EPID based and has the ability to perform automatic self‐integrity check of LINAC performance. Meanwhile, most of the previous works were developed and validated for one specific LINAC system and only covered photon beams with flattening filters (FF). For the MPC tool, only high‐end Varian LINACs are equipped with it and the tool is not available in low‐end LINACs from Varian and other vendors. In addition, MPC was developed by Varian and tightly integrated with their LINACs. However, it is often more desirable to have a daily QA tool that is independent of the LINAC vendor to facilitate the intersystem comparisons.^24^ In this study, both FF and flattening filter‐free (FFF) photon beams are covered, and the robustness of the proposed EPID‐based QA method was evaluated across different LINAC vendors and models. To be more specific, a rapid EPID‐based daily QA tool entitled DQRT (Daily QA Research Tool) was developed in this work, and its reliability was verified using multiple Elekta (Synergy, VersaHD) and Varian (TrueBeam) LINAC systems. The tool can be used to evaluate dose constancy, beam quality (BQ), flatness (F) and symmetry (S), center of field, and field size accuracy. The proposed method was used to perform daily QA for both the 6‐MV FFF and FF photon beams. In addition to the cross‐platform and multienergy features mentioned above, the theory of this tool is straightforward and robust which make the tool have a strong reliability. In this study, we first demonstrate the physical principle and parameter calculation process of DQRT, then the stability and accuracy are assessed by comparing with other devices. At last, the clinical performance of DQRT was compared with DailyQA3 and Beamcheck in different linacs, which showed that the DQRT is more convenient for daily QA of Linacs.

## 2 METHODS AND MATERIALS

### 2.A Calibration of EPID

In order to ensure the accuracy and precision of DQRT, we performed calibration of the EPID system of each LINAC via the following procedures.

For the Elekta LINAC, the iViewGT system was used. The MV detector of the iViewGT system has a fixed source‐to‐skin distance (SSD) of 160 cm and can only move along the longitudinal and lateral directions. This EPID has been calibrated using the following procedures^25^: First, the mechanical accuracy of EPID was verified. The longitudinal movement was operated with a handheld controller in order to make sure that the reduced‐speed, isocenter pause and longitudinal limit work correctly. Next, the MV detector was moved to the isocenter position, and the mechanical pointer on the longitudinal scale was checked to make sure it points to the isocenter mark within ± 1 mm. For the lateral direction, the same validation step was performed. Then, the offset correction and the first radiation synchronization calibration were auto‐executed by the iViewGT system. Next, a gain calibration was performed at the zero degree gantry angle and collimator angle, the maximal dose rate, 26 cm × 26 cm field size, and an exposure dose of 100 MU. After the gain calibration, a bad‐pixel map was applied to help correct those pixels known to give inconsistent responses. Finally, a second radiation synchronization calibration was performed in order to make sure that the image has a clear contrast. After these steps were completed, image scaling was executed starting from an exposure image with a field size of 15 cm × 15 cm and an exposure dose of 10 MU. Then the horizontal size of the exposed field was measured used the iViewGT measure tool. After being divided 150 by the horizontal size, the result was set to the “HorizMMPixel” value that stored in the field with a file name of sri.ini.

For the Varian LINAC, one TrueBeam system equipped with an amorphous silicon (aSi‐1000) portal imager was used. The aSi‐1000 imager consists of 1024 × 768 pixels, each with a pixel size of 0.39 × 0.39 mm^2^. The aSi‐1000 was calibrated by creating the corresponding dark field (DF) image and flood field calibration file pairs. The DF image was acquired without radiation and was averaged over a series of measurements to provide the pixel offsets. The flood field image was acquired by irradiating the detector panel with an open field covering the entire imager. The measurements were averaged over a fixed number of frames to determine the mean difference in individual pixel sensitivity. Dosimetry calibration was applied based on the recommendation from Varian; the beam diagonal profile measured at D_max_ in water for the 40 cm × 40 cm open field was used to scale the off‐axis pixel response, and dose normalization is performed to set the calibration unit (CU) of the portal dose equal to the clinical dose unit (cGy). Finally, a pixel correction map generated from the approved DF and flood field images was applied to the TrueBeam system.^26^

### 2.B Image acquisition and processing

All measurements of the Elekta LINACs were carried out using the EPID in its default position without any additional build‐up. The source‐to‐detector distance (SSD) was kept at 160 cm. The detector signals were read out with a fixed integration time of 250 ms for each image frame. After the exposure, all images were added up over the entire exposure time and integrated into a 64‐bit buffer. At last, an arbitrary scaling factor is included to be able to store the signal data in a 16‐bit format and a final portal dose image with *.tif format was produced after the standard image processing procedures was performed by iViewGT automatically.

The measurements of TrueBeam were performed in the clinical mode and the integrated images were acquired when the test beams were executed. The SDD was kept at 100 cm without any other additional build‐up in all tests. The “PortimageIntegrated” mode designed for dosimetric application was selected to get the integrated image. In this mode, the dose for per frame image keeps constant and the image frame readout time is approximately 110 ms. The data flow began with the beam being triggered by the Truebeam supervisor module. Next, the MV image acquisition module was triggered by the MV imager and the detector began to acquire the frames. The digitized images were processed and corrected by an XI software based on the stored calibration set in the software. At last, the image encoded in a standard DICOM RT image was exported from the imaging workstation.^26^

### 2.C Test and evaluate methods

Based on the recommendations of AAPM TG‐142,^27^ the DQRT mainly focuses on the stability of output, BQ, F and S, center of field, and field size.

For the first step of the proposed QA procedure, two EPID images were acquired when the gantry angle was at zero: one (I1) with an open field and the other one (I2) with a 60° wedge. Both images were obtained with 10 cm × 10 cm field size and 100 MU. When the DQRT was first applied to a LINAC, the LINAC was adjusted to its best state, and the values of monitored parameters mentioned above were obtained by DQRT at this time served as the benchmark data. In each work day, I1 and I2 images were reacquired using the same setup mentioned above. The output, BQ, F and S, center of field, and field size of each day were obtained from I1 and I2 and compared with the benchmark data. If any inconsistency of the major parameters of LINAC was observed, or if status such as mechanical and image calibration, version of control software, etc., of the EPID were changed, the LINAC should be investigated and the benchmark data need be reacquired. If none of the above has happened, the benchmark data are reacquired each year.

Mean grayscale pixel value of a 10 mm × 10 mm central region in each image obtained by DQRT is defined as μ. The stability of output was evaluated based on μ value of the image I1. The profiles along X and Y axes were calculated from the average grayscale values of ten rows in the field center. Next, the field edges were determined by points whose first derivative values along X or Y profile are maximal and the second derivative values of them are zero. Then, the field sizes along the X and Y directions were calculated and the intersection of the field diagonal lines was considered as the field center. For the F and S, they were quantified based on the X and Y profiles of I1. Formulas 1 and 2 are the formulas used to calculate F and S. The BQ, which could be reflected by wedge factor W, was determined from μ_wedge_ and μ_open_ using Formula 3.

(1) $$F=\frac{D_{\text{max}}‐D_{\text{min}}}{D_{\text{center}}}\times 100\text{\textbackslash{}\%}\;$$

(2) $$S=\frac{\text{max}\left(\left|D_{\text{left}}‐D_{\text{right}}\right|\right)}{D_{\text{center}}}\times 100\text{\textbackslash{}\%}\;$$

(3) $$W=\frac{\mu_{\text{wedge}}}{\mu_{\text{open}}}$$

**Fig. 1:**
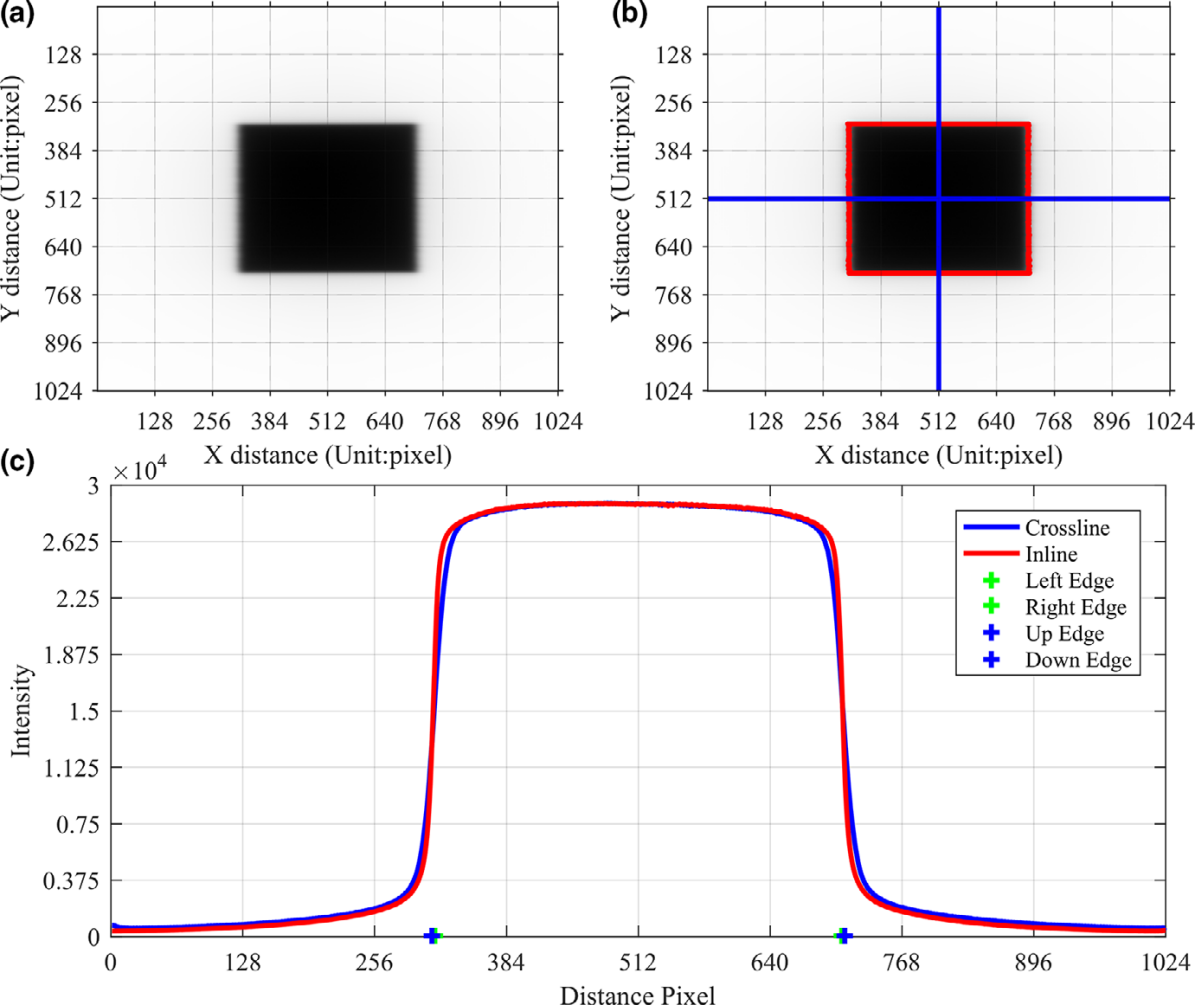
Readout step of the DQRT for 10 cm × 10 cm open field. (a) The field measured by EPID (b) Field edge detected by the DQRT (c) X and Y profile and its edge points.

In the Formulas above, $D_{\text{max}}$, $D_{\text{min}}$, $D_{\text{center}}$ represent the maximal dose, minimal dose, central dose, respectively; $D_{\text{left}}$,$D_{\text{right}}$ are the dose of two points which are symmetric about the field central axis. All of the points above are selected from X or Y profile and within 80% region of the field size. The readout step of the DQRT for the field is illustrated in Fig 1.

### 2.D Short‐term stability of EPID

Two sets of tests, referred to as A and B, were performed in the Synergy system to verify the short‐term repeatability. For each set, the EPID was irradiated by a 10 cm × 10 cm open field and an exposure of 100 MU for ten times, generating ten images. Note that the MV detector panel was retracted and then extended between each measurement for test B while no operation was applied to test A. Field size, F and S and coordinates of beam center were analyzed for both A and B. These tests were also assessed on the TrueBeam LINAC of our unit.

### 2.E Long‐term stability of EPID

Position and dosimetry stability were evaluated by the methods proposed by B.W.King.L etc.^28^ Two images were obtained for the 6 MV 10 cm × 10 cm field each day, one with a collimator angle 90° and the other one with 270°. The mean center of the two images was set as the beam center. A total of 45 measurements were performed during 9 weeks in each LINAC separately. The variation of the beam center was used to characterize the stability of the positioning system.

As for the dosimetry stability, the center dose of a 10 cm × 10 cm image with 100 MU exposure measured by EPID at 100 cm SSD, was compared with the result measure by an ion chamber (IBA FC65‐G, IBA, Dosimetry GmBH, Schwarzenbruck, German) in a one‐dimensional (1D) water tank during our weekly QA. The measurements were performed for six consecutive months on the Truebeam and Synergy systems and for 2 months on an Axesse system.

### 2.F Linearity of the EPID outputs

A set of 27 beams was measured with the Synergy system to validate the linear relationship between the exposure and μ. For all of the beams, the experiments were repeated four times in order to reduce the statistical uncertainty. The field size was set to 10 cm × 10 cm for these experiments. The MU for the 27 beams are 1, 2, 3, 4, 5, 6, 7, 8, 9, 10, 20, 40, 80, 95, 96, 97, 98, 99, 100, 101, 102, 103, 104, 105, 150, 200, and 300. A set of eight beams that have the same geometry to the 27 beams was measured with the TrueBeam system, with exposures of 10, 20, 50, 80, 100, 200, 300, 600 MU, respectively. The μ value calculated by the DQRT from image acquired by the EPID system for each beam was plotted against MU, and a linear fitting of the curve was performed. The R^2^ value of the linear fit was calculated and used as the figure‐of‐merit for linearity.

### 2.G FFF photon beam

For Synergy and TrueBeam systems used in this research, their detector panels do not support the high dose rate mode. Therefore, only the 6‐MV FFF photon beam of VersaHD was studied. The profile of the FFF ray has a high value in the middle area and low value in the peripheral area which is more sensitive to the position variation. Therefore, the influence of the setup position accuracy to output and BQ was investigated using the following method: when DQRT reads the output from EPID image, errors from 1 mm to 10 mm with 1 mm interval, 15 mm, 20 mm, and 30 mm were artificially introduced to the read position to field center’s left, right, gantry (G), and the target (T) direction respectively. For comparison, the tests above were also applied to the 6‐MV FF X ray. For FFF photon beam, except the beam F, the same parameters were monitored.

### 2.H Validate DQRT

During a period of 2 weeks, a 6‐MV photon beam with 10 cm × 10 cm field size, 100 MU exposure, and a SSD of 100 cm was executed two times by the DQRT and three times by the FC65‐G detector per day on the Truebeam LINAC system. For the detector measurement, the FC65‐G was initially set on the beam center axis with 5 cm distance under the surface to measure the output. Then the measured depth was adjusted to 20 cm and 10 cm to monitor BQ in formula.4

(4) $$\text{BQ}=\frac{D_{20\;\text{cm}}}{D_{10\;\text{cm}}}$$

A series of fields with sizes ranging from 9.4 cm × 9.4 cm to 10.6 cm × 10.6 cm (0.2 cm step size) were measured two times both by the IBA Matrixx and the DQRT. The exposure for all of the measurements was 300 MU. For the Matrixx, SDD (Source Detector Distance, SDD) = 100 cm and SSD = 95 cm were chosen, and for the DQRT, the default position mentioned in 2.2 section was used. The FermiFit interpolation method and 0.01 cm distance between rows were selected in the OmniPro IMRT software to analyze the field size measured by Matrixx. The field size intervals in the test series measured by DQRT and Matrixx were compared to the actual values.

The DQRT was implemented in a Synergy LINAC for 2 months and a TrueBeam system for 1.5 month. During the 2‐month time window, the QA data on the dose constancy, BQ, F and S, center of field, field size for the 6‐MV FF photon beam were collected. In order to compare against some daily commercial solutions, parameters mentioned above were measured by DailyQA3 (Sun Nuclear Co., Melbourne, FL) for the Synergy and by QABeamCheckerPlus (Standard Imaging Inc., Middleton, WI) for the TrueBeam. For the 6‐MV FFF photon beam, a VersaHD system was monitored by both DQRT and QABeamCheckerPlus for 2 months. Note that BQ was not studied for TrueBeam because the QABeamCheckerPlus does not support this parameter.

## 3 RESULTS

### 3.A Short‐term stability of EPID

**Fig. 2:**
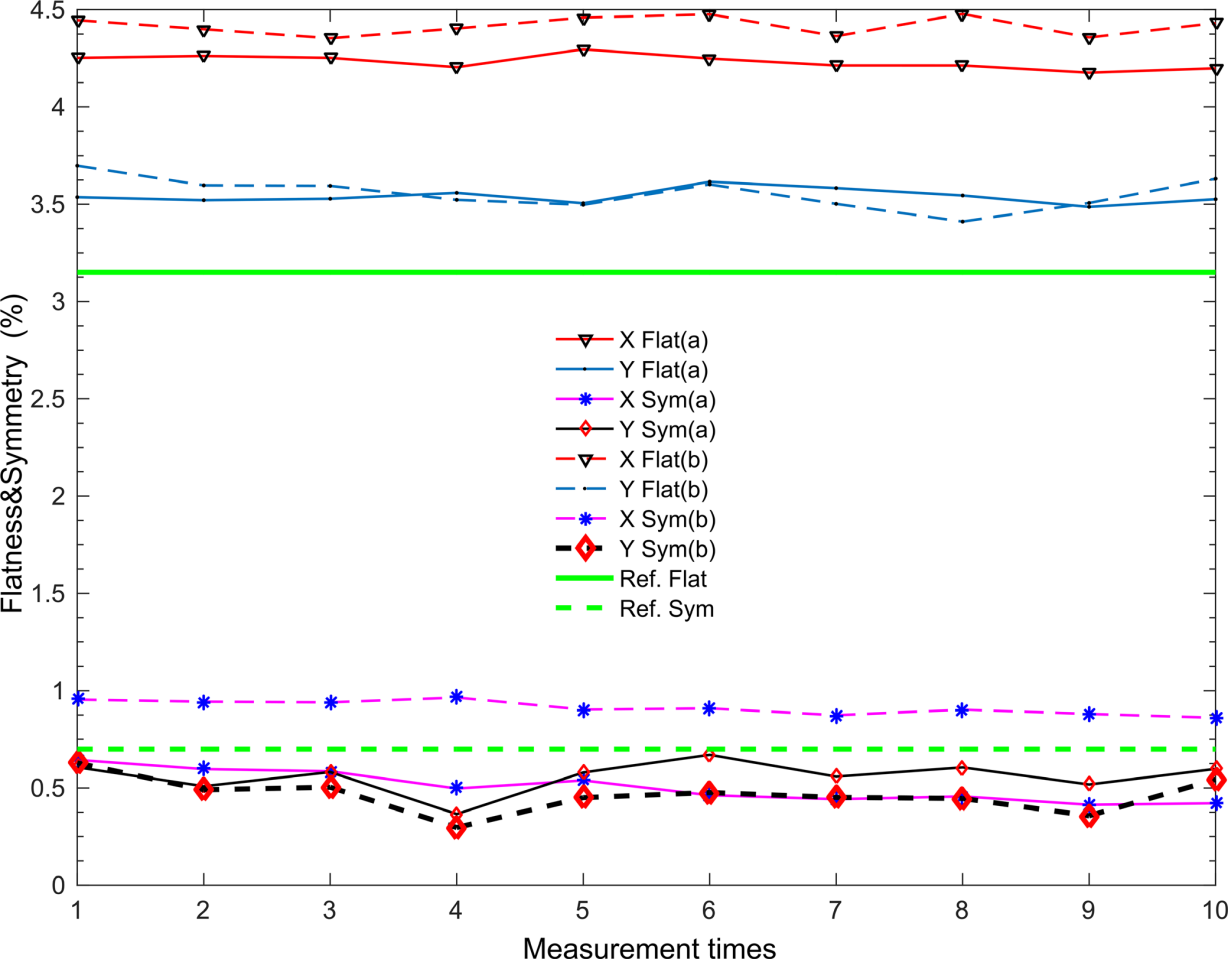
Flatness and Symmetry measured by the DQRT in repeatability tests. (a) Group A, (b) Group B. “Ref. Flat,” and “Ref. Sym” are flatness and symmetry value from Synergy commissioned data which measured with 10 cm × 10 cm field and 100 cm source to surface distance, 5 cm depth under the surface.

**Fig. 3:**
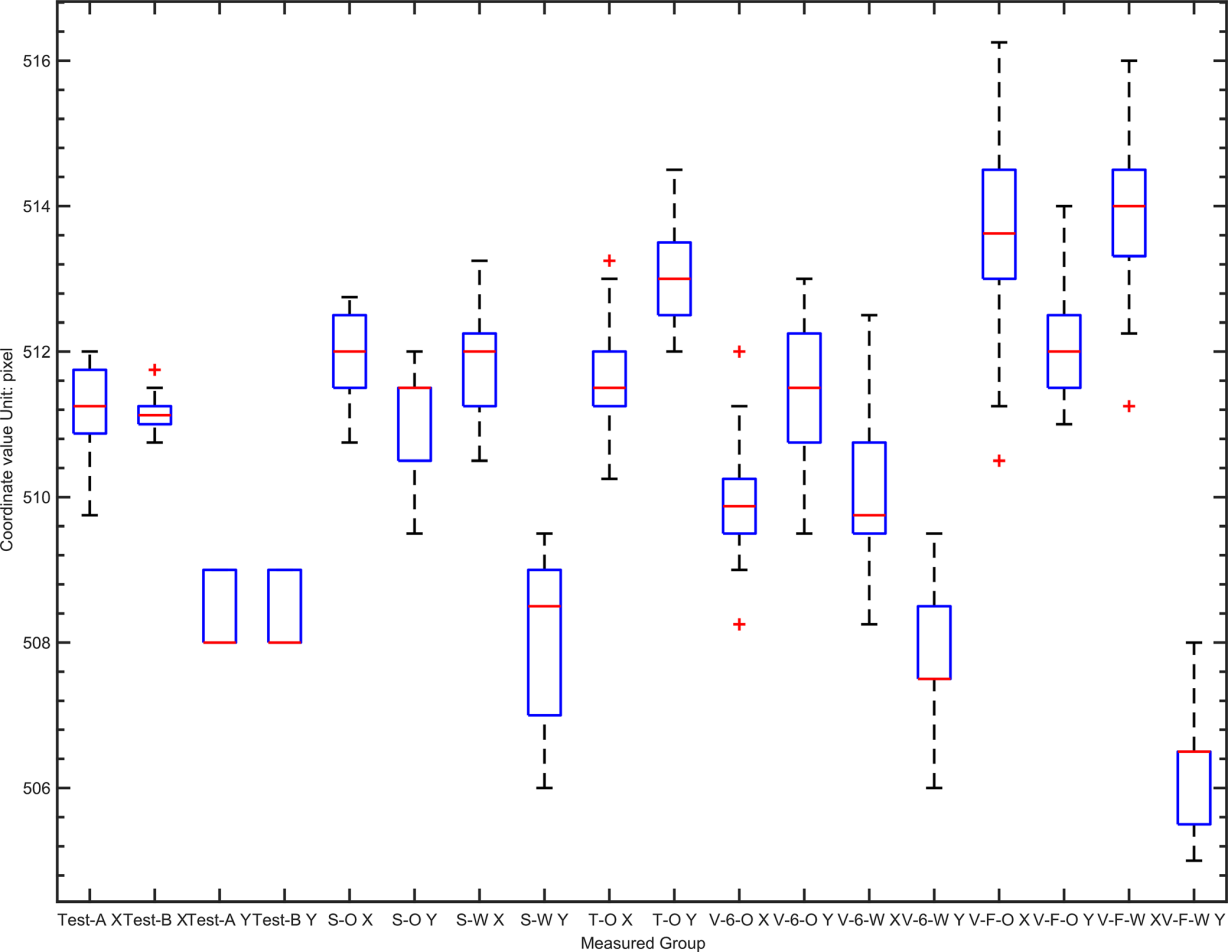
Beam center distribution in all the tests. A and B correspond to the repeatability test in 2.3; “S,””T,””V,””O,””W,””X,””Y,””6,”and “F” represent the Synergy, TrueBeam, VersaHD, Open field, Wedge field, left‐right (LR) direction, gantry‐target (GT) direction, 6 MV beam, 6 FFF MV beam, respectively. For example, VFWX means LR direction coordinate of the beam center of a 6‐MV FFF photon field with wedge measured in VersaHD LINAC system. For GT direction, coordinate of TrueBeam, a value 128 is added.

The measured field sizes along the x and y directions range from 0.505 mm to 0.507 mm for test A and 0.508 mm to 0.761 mm for test B. The difference between the two test groups is only 0.003 mm (test A) or 0.254 mm (test B). Either in the x or y direction, there is a difference less than 0.390 mm for the center of the field measurements of the two test groups and a difference less than 0.05% for F and S in each direction between two test groups. Therefore, there is no significant difference between the two groups of measurements. Detailed results of all the repeatability tests can be found in Fig. 2 and Fig. 3.

### 3.B Long‐term stability of EPID

**Fig. 4:**
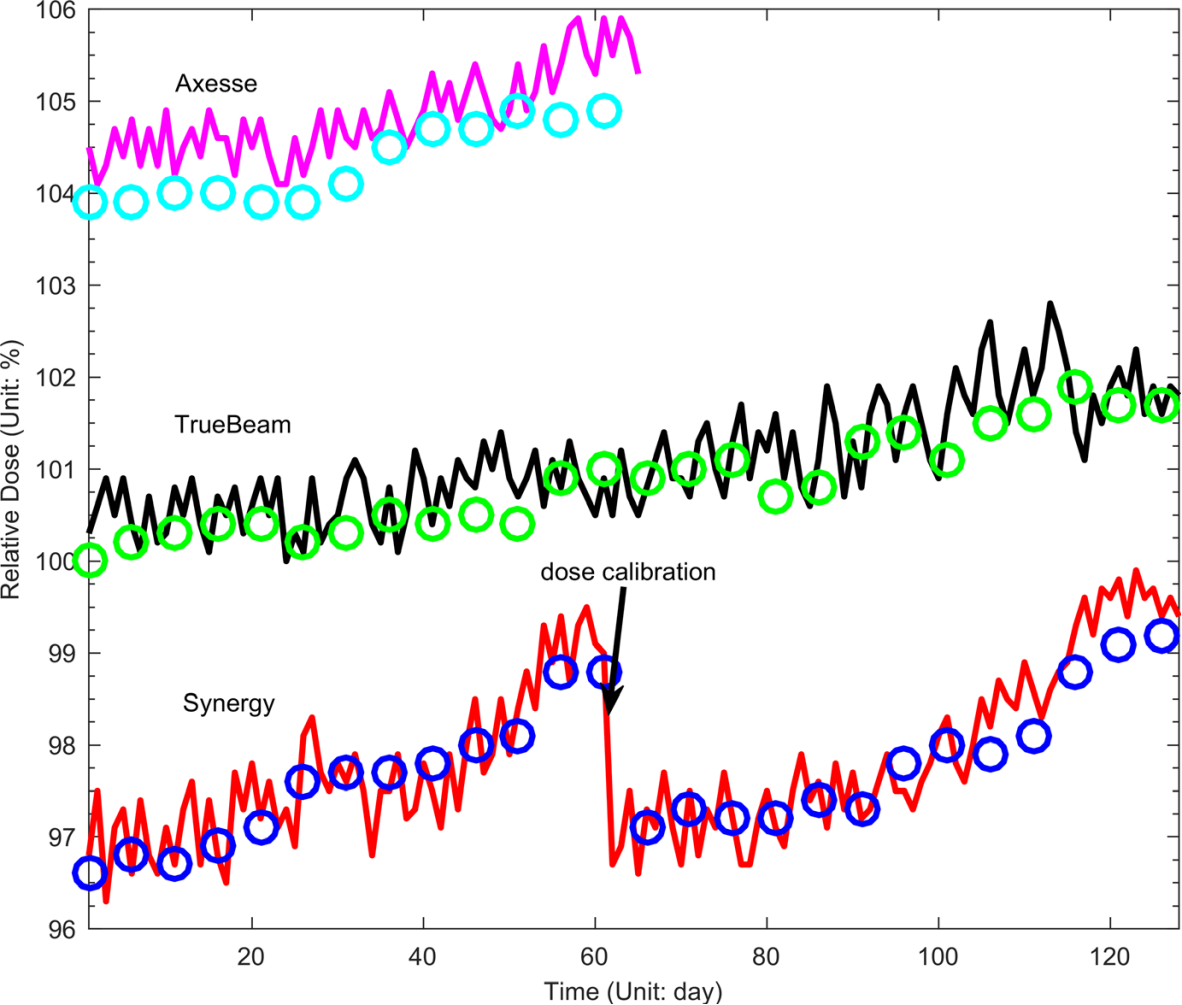
Measured EPID central values (Circles) compared with output values of LINACs measured by FC65‐G in one‐demission water tank (lines).

The variation of radiation center of each LINAC measurement by the EPID was shown in Table 1. To all of the LINACs, the mean position coordination value of EPID central axis variation were all less than 1.5 pixels. They show that all of the EPIDs could be positioned very accurately over a long period of time. The comparison of image central axis measurements between EPID and FC‐65G along with the time was shown on Figure 4. Differences between ion chamber and DQRT measured during this period are −0.16% ± 0.28%, −0.20% ± 0.37%, −0.62% ± 0.22% respectively for Synergy, TrueBeam, and Axesse. As can be seen from the figure, values measured by EPID closely match the detector values.

**TABLE 1:** Mean offset between the EPID and beam center measured over time.

LINAC	Cross‐plan		In‐plan	
	mean (mm)	SD (mm)	mean (mm)	SD (mm)
Synergy	0.0408	0.09	−0.1031	0.11
Axesse	−0.1839	0.06	0.3981	0.18
TrueBeam	−0.4865	0.05	−0.0567	0.08

### 3.C Linearity of the EPID outputs

For the Synergy system, the relationships between μ and the monitor unit (MU) for all of the four measurements are linear. For the mean value of μ of the four measurements, the fitting function was obtained, as shown in formula 6, when all grayscale values were normalized to the value of 100 MU. The mean R^2^ value of the four fitted curves is 0.998. For the TrueBeam system, the fitting function is shown in formula 7 and R^2^ value is approximately equal to 1. These data demonstrated that the μ is proportional to the exposure for a given 10 cm × 10 cm open field beam, so the exposure can be estimated from μ.

(6) $$\mu /\mu_{100\text{MU}}=1.0540\times \text{MU}‐5.2985$$

(7) μ=1.0012×MU‐0.1766

### 3.D FFF photon beam

**Fig. 5:**
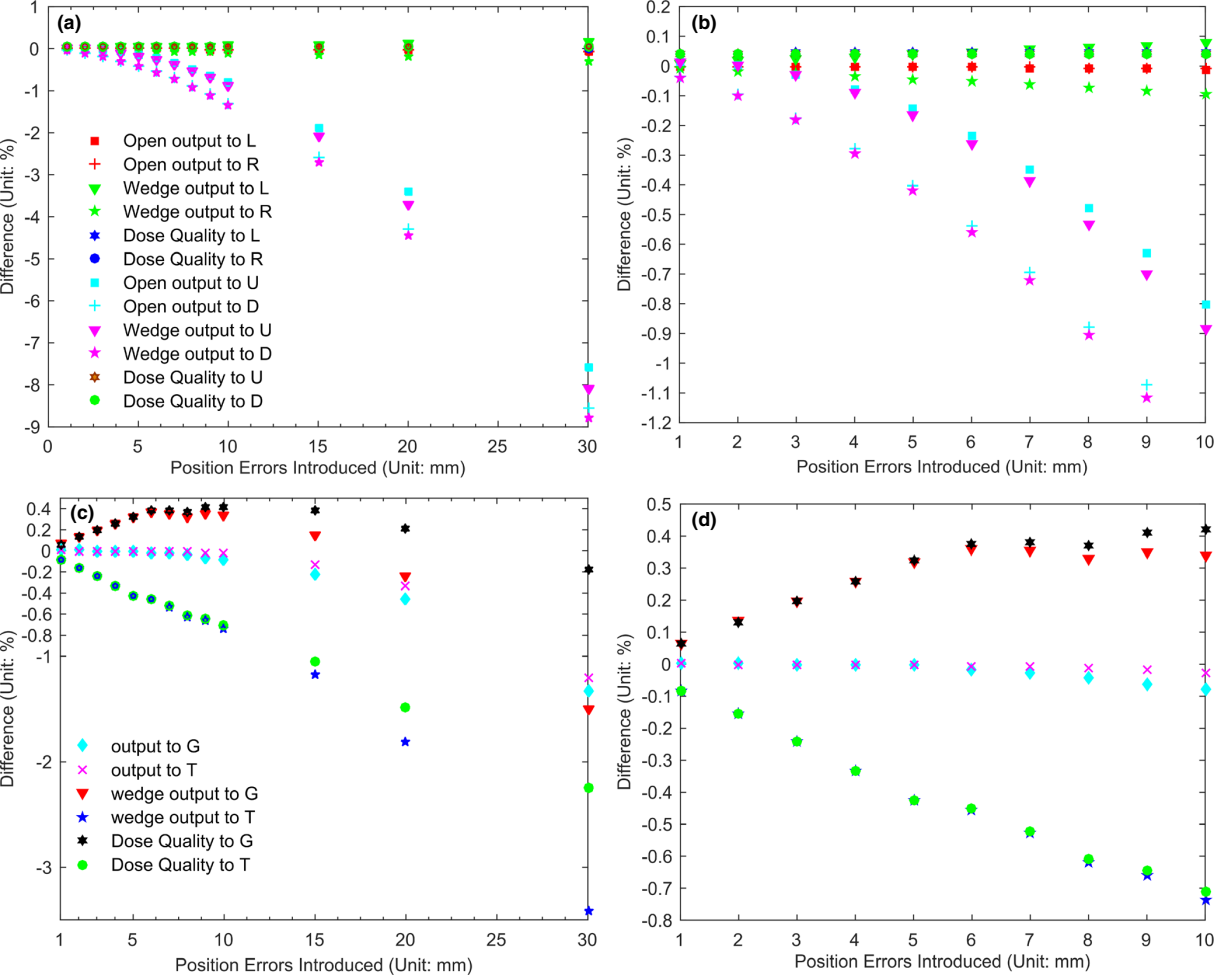
Output and beam quality deviation by the artificially introduced position errors to different directions. U, D, L, R represent the gantry (G), target (T), left, and right direction respectively. (A) and (C) are results of 6 MV FFF and 6 MV photon beams. (B) and (D) are a part of (A) and (B) with x axis varied from1 mm to 10 mm.

For all of the open and wedged fields, whether the position error is artificially introduced to its left, right, G, or T direction, the measured output deviations ranges between −1.36% and −0.08% and BQ deviations are all smaller than 0.08% with the error value increasing from 1.00 mm to 1.00 cm. When the error value is 1.50 cm, the measured output deviation is about −2.81% [(Figs. 5(a) and 5(b)]. Results of the FF photon beam are shown in [(Figs. 5(c) and 5(d)]: BQ varies from −0.71% to 0.42% and output varies from −0.73% to 0.25% when the introduced position error deviation increases from 1.00 mm to 1.00 cm. When this error deviation reached 1.50 cm, BQ and output deviation reached −1.11% and −1.23% respectively.

### 3.E Validate DQRT

**Fig. 6:**
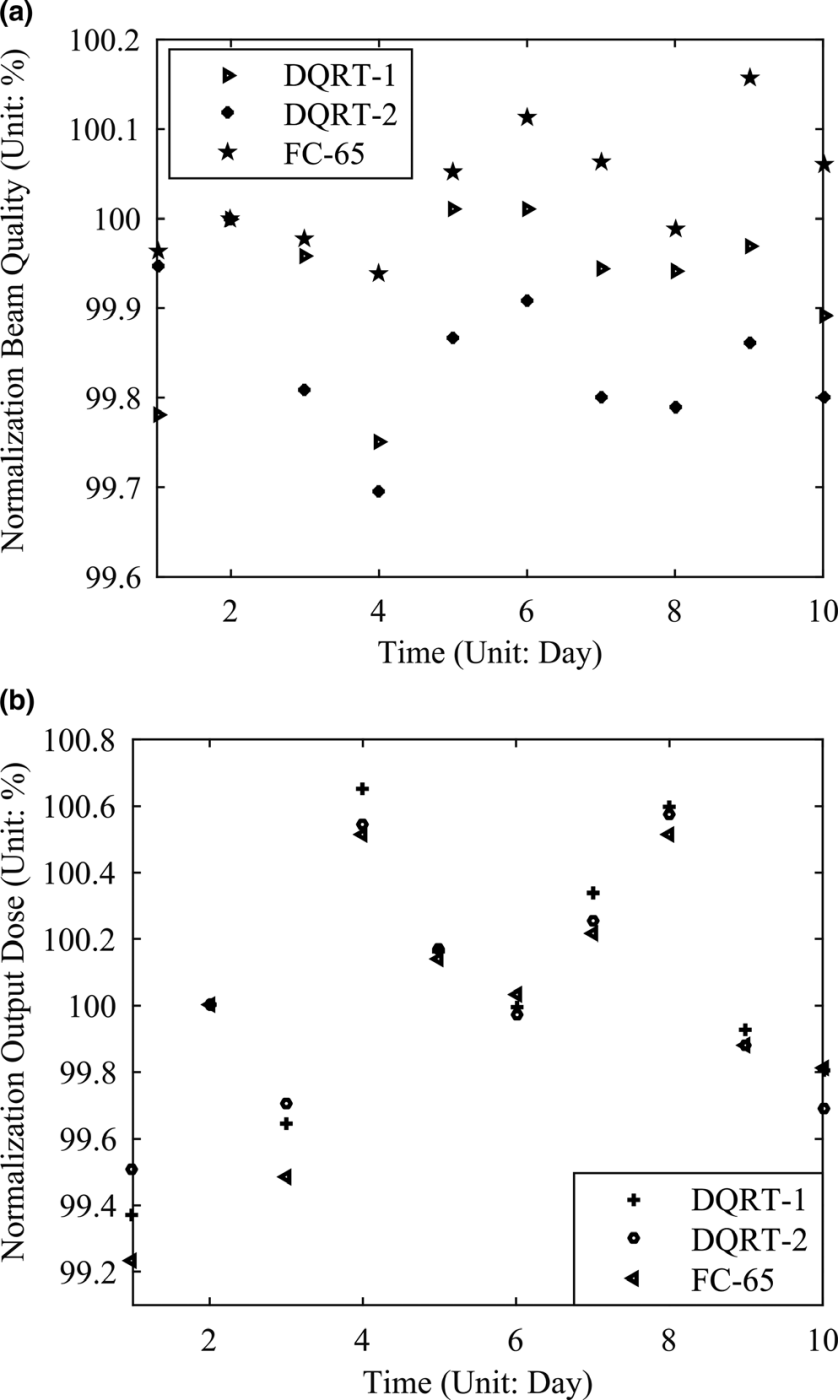
Normalized beam quality (a) and output (b) of the TrueBeam 6 MV X ray measured by DQRT and FC65‐G during 2o weeks.

Normalized BQ values measured by FC65‐G detector are in 99.94%–100.16% and normalized W values bilateral measured by DQRT are in 99.75%–100.01%, 99.69%–100.00%. Normalized output obtained by DQRT with two times measurement and FC65‐G farmer detector with one time measurement distribute in 99.23%–100.52%, 99.37%–100.65%, 99.51%–100.58%, separately (Fig. 6). The size variation between two adjacent fields in the test series are all within 1.56 mm–2.34 mm measured by DQRT and within 1.80 mm–2.30 mm, 1.90 mm–2.10 mm measured by Matrixx along X and Y directions. The discrepancy between them distribute in −0.59 mm to 0.34 mm to the crossline and −0.34 mm to 0.34 mm to the inline. All the results are consistent with the value (2.00 mm) set before (Table 2).

**TABLE 2:** The adjacent field size variation measured by DQRT and Matrixx two times each. All the unit in this table are mm.

change in field size	DQRT1		DQRT2		Matrixx1		Matrixx2	
	X	Y	X	Y	X	Y	X	Y
2 (96–94)	2.34	1.95	2.34	1.56	1.90	2.10	1.90	2.10
2 (98–96)	1.56	1.56	1.56	1.95	2.00	2.00	2.30	1.90
2 (100–98)	1.56	2.34	1.95	2.34	2.30	2.00	2.00	2.10
2 (102–100)	1.95	2.34	1.56	1.95	2.30	1.90	2.30	1.90
2 (104–102)	2.34	1.56	2.34	1.95	2.00	2.00	2.00	2.00
2 (106–104)	1.56	2.34	1.56	2.34	1.80	2.00	1.80	2.00

**Fig. 7:**
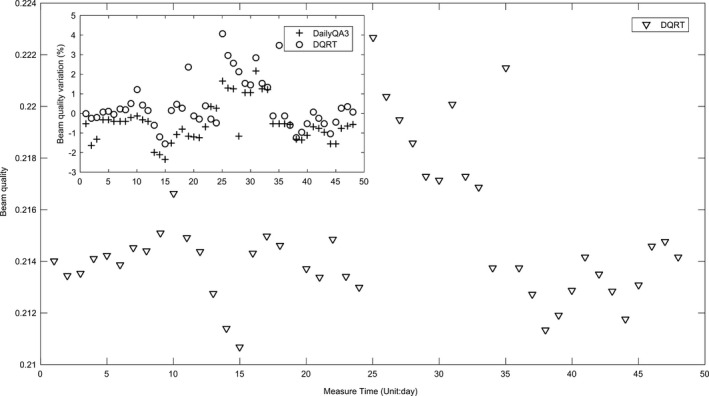
Beam quality of Synergy measured by DailyQA3 and W value measured by DQRT with different measurement time.

**Fig. 8:**
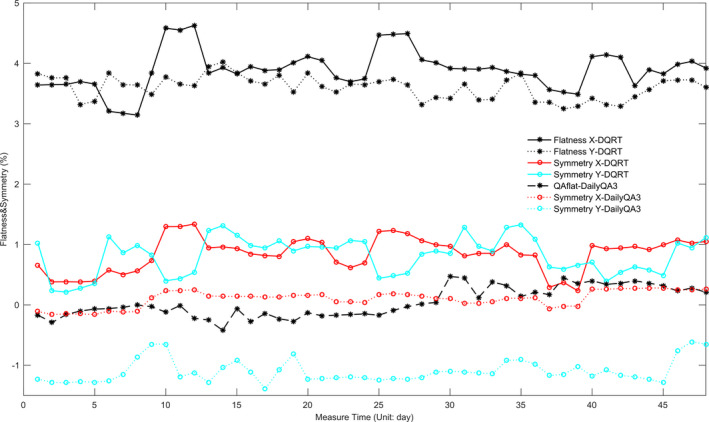
Flatness and Symmetry measured by DQRT and DailyQA3 changed with different measurement time.

For the Synergy system, the ratios between the outputs detected by DQRT and the benchmark distributes between 0.980 and 1.018, which is consistent with the results measured by DailyQA3 (0.978–1.020). The ~RT (−1.82% to 4.13%) is larger than that (−2.41% to 2.22%) of DailyQA3 (Fig. 7) and a strong correlation was found between them ( Pearson’s correlation coefficient is 0.71). X and Y field size of the open field detected by DQRT are within 100.547 mm–101.309 mm and 101.000 mm–102.070 mm, respectively. However, the results of the same field measured by DailyQA3 range within 100.000 mm–100.100 mm and 100.007 mm–100.150 mm, respectively. The trend of the F and S of the open beam measured by the DQRT and DailyQA3 is consistent, and all of the measured results are within tolerance. The F defined in DailyQA3 was calculated by the dose both of the X and Y direction, so there is only one set of values. Because the definition of F is different, F value measured by DQRT are 3–4 percent higher than DailyQA3. To the S, the difference reduced to 1% for crossline and 2% for inline (Fig. 8). The range of S and F value corresponding to X and Y direction measured by DQRT are 0.85 ± 0.28 and 0.81 ± 0.31, 0.39 ± 0.00 and 3.90 ± 0.33 respectively. For these values measured by DailyQA3, the distribution are −1.10 ± 0.19, 0.10 ± 0.13 and 0.04 ± 0.24 (Flatness results measured by DailyQA3 are direction‐free). The position of the detector pixel corresponding to the beam center ranged within 511–513 along the X direction, 510–513 along the Y direction.

**Fig. 9:**
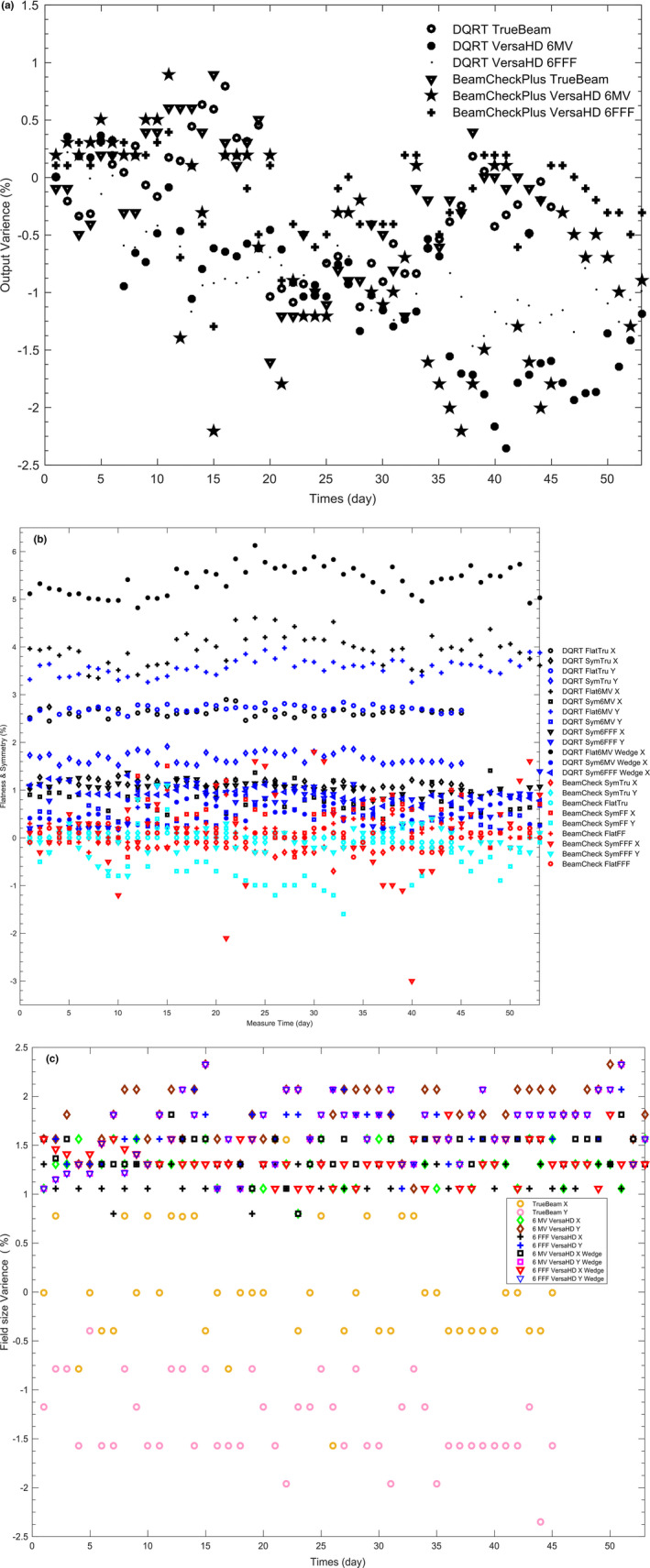
Clinical test result of 6 MV photon beam of VersaHD & Varian system and 6 MV FFF photon beam of VersaHD system. QABeamCheckerPlus is used as a reference. (a) Dose output. (b) Flatness and Symmetry. (c) Field size. Both left‐right (X) and GT (Y) direction are shown in (b) & (c).

For the 6‐MV photon beam of TrueBeam, VersaHD system and the 6‐MV FFF photon beam of VersaHD system, the measured dose constancy, beam size, F and S by DQRT all have the similar values to the results measured by QABeamCheckerPlus at the same time. Output differences between BeamCheck and DQRT to Truebeam, 6MV VersaHD, 6FFF VersaHD are −0.6% to 0.7%, −1.3% to 0.7%, −0.9% to 1.1%, separately [Fig. 9(a)]. S differences measured by the two devices are −1.1% to 3.3%, 1.5%–1.8% corresponding to X and Y directions of TrueBeam. For 6MV and 6FFF X beam of VersaHD, the differences distribute in −0.6% to 1.4%, −0.2% to 1.3%, and −0.5% to 3.2%, −0.8% to 1.2% [Fig. 9(b)]. Field sizes deviation measured by DQRT is in the range of −1.6% to 0.7% and −2.3% to −0.55% to X and Y direction of TrueBeam. For 6 MV, 6FFF open field of VersalHD, deviation values mentioned above distribute in 1.4%–1.8%, 1.9%–2.4% and 0.8%–1.3%, 1.1%–1.7%. As for the wedge field, the range become 0.8%–1.8%, 1.1%–2.4% and 1.1%–1.8%, 1.1%–2.4% [Fig. 9(c)]. For each test series, the change of beam center position is within three pixels (Fig. 3).

## 4 DISCUSSION

As both Clivio et al.^22^ and Sun et al.^17^ showed, EPID‐based daily QA could monitor much more LINAC parameters than conventional QA device with the same or shorter amount of time. Previous works on EPID‐based daily QA mainly focused on a particular type of LINAC and FF photon beams.^23^ In this manuscript, a more comprehensive multivendor and multibeam type study was performed to evaluate the proposed EPID‐based daily QA tool. It took only 2 min for the tool to measure all daily QA parameters while it required 5 min for QABeamCheckedPlus and 8 min for DailyQA3 to accomplish the same set of QA tasks.

Concerning the lack of enough independence, cost‐effectiveness, and mechanical insufficiency of EPID, Sasa Mutic et al.^24^ have given their suggestions and our results also give reasonable explanation. Position error test shows that, the BQ error is less than 1.08% when the error is no more than 15 mm for both 6 MV FF and FFF photon beams [Figs. 5(a) and 5(b)]. It means that the EPID setup variation between each time is too subtle to have any effect on the final results. Furthermore, only one computer is needed to use DQRT to perform daily QA, which is more cost‐effective than the conventional approaches.

The short‐term and long‐term position, dosimetry, response stability of EPID, and ghosting, saturation effect greatly affect the DQRT reliability. The maximum dose rate was restricted to avoid the saturation effect and the ghosting effect was eliminated by increasing the interval time between each two measurements to more than 2 min. Because the results of repeatability test A and B are associated with position, dosimetry, and response stability of EPID, short‐term stability of the three factors could be evaluated by the result of A and B at the same time. For the long‐term stability, position and dosimetry factors were evaluated in section 2.5 and the response stability was neglected, because the response correction was done automatically by EPID in each measurement and the short‐term stability could guarantee its reliability. There is no fluctuation of the performance and condition of EPID during this research, which also indicates that the stability of EPID is sufficient for daily QA.

For FFF photon beam, the high‐dose rate and the distribution characteristics are major challenges for the time resolution and setup accuracy of EPID detectors. In research, a maximal dose rate 1400 MU/min and SDD 160 cm were selected when the FFF photon beam tests were performed, which could have bypassed the saturation effects.^29^ As regardse the effect of FFF photon beam distribution, the sensitivity of DQRT to the position errors has ruled out the concern.

For the repeatability test, one problem should be addressed: the maximum variation of output measured by EPID is 0.10% for the repeatability measurement, which is much larger than the fluctuation of the ionization chamber, such as 0.03% for FC65‐G chamber obtained by ten times repeatability measurement. Therefore, the larger fluctuation of EPID may introduce a larger error than the recommendation of TG142. In order to avoid this, a tighter tolerance ± 2.5% is selected as the dose constancy standard in our study.

Another problem revealed by the results is, when the exposure is small, parameters such as field size, F and S fluctuated strongly during the repeatability measurement. For the Synergy LINAC, the fluctuation scope of the parameters detected by DQRT is close to 5% when the exposure is smaller than 10 MU, and the scope decreased to 1% when the exposure increased to 10 MU–80 MU. When the exposure becomes larger than 80 MU, the scope reduces to less than 0.1%, and it can be considered that the measurement stability of each parameter no longer changes with the increase of exposure. Therefore, in clinical practice, exposure for daily QA using EPID is suggested to be greater than 80 MU. However, too much exposure would have a negative effect on the EPID detectors, so a larger number of MU is not recommended. Considering the above reasons, 100 MU is selected as the exposure in DQRT.

Results of clinical size measurements show that, field sizes of Synergy and VersaHD measured by DQRT are slightly above the set values, which are the same to the calibration results of Autocal^TM^ (IBA, Scanditronix Wellhöfer GmbH, Bahnhofstraße 5, D‐90592 Schwarzenbruck). For Elekta LINAC, position of Beam Limiting Device (BLD) is controlled by “Gain” and “Offset” in different positions. Therefore, when the field size formed by BLD is too small (about less than 20 cm), the actual position of BLD would be larger than the set value. Otherwise, the actual position of BLD would be smaller than the set value.^30^

Another issue needs to be addressed is that, for the DailyQA3 and the QABeamCheckerPlus, the definitions of BQ, F and S are different from those used in the DQRT. As a result, the measured values between them have obvious difference, but the general trend of these parameters is consistent [Figs. 7 and 8], which means that the DQRT has the same accuracy and reliability as the DailyQA3 and QABeamCheckerPlus. In DQRT, though the measured values of the parameters are not confirmed with the actual values, they are also used to reflect the trend of corresponding parameters. This could be done because the parameters calculated in DQRT have a stable relationship with the parameters in reality, and because the daily QA in clinical practice focuses primarily on the data stability.

The research suggests that the DQRT could be used for daily QA of electronic beam. For electron BQ measurement, a glass with appropriate thickness could be chosen as a substitution of the wedge, and the glass can be placed on the surface of EPID detector. Other tests for electron beam are similar to the FF photon beam.

In addition, a region of 40 pixel × 40 pixel located at the field center is selected to measure the output in this study, which is the same with Sun etc. al.^24^ For different LINACs, the size of the pixel may be different. Hence, in order to keep the balance between precision and anti‐interference, appropriate region size should be chosen for different systems.

## 5 CONCLUSIONS

Several EPID‐based QA methods have been proposed, including in‐house tools and commercial products, such as MPC. However, these schemes have some disadvantages, such as generality and independence inadequate. This work demonstrates that the EPID‐based tool (DQRT) is capable of daily QA for multiple LINAC systems with and without flattening filter and is suitable for each type of LINAC. At the same time, it can monitor more aspects of the LINAC performance than conventional devices within a shorter period of time with stable and reliable results. In addition, the only additional equipment required for this method is a conventional computer, and thus the proposed method is very cost effective. In summary, DQRT could serve as a low‐cost and highly efficient daily QA tool. The DQRT is vital for detecting potential mechanical and dose issues due to the increasing demand of integration and automation of QA works.

## Author Contributions

Yangguang Ma and YueXin Guo proposed the original notion of the research and Yangguang Ma wrote the manuscript, Xuemin Wang and Rizhen Mai designed and implemented the algorithm. Tao Wang and YunTong Pei designed the experiments and implemented the data generation process. Shuaipeng Liu carried out experimental work. All the authors reviewed the manuscript.

## Conflict of Interest

The authors report no conflict of interest. The authors alone are responsible for the content and writing of the paper.
